# Identification of *BRCA2* Likely Germline Pathogenic Variants in Patients with Multiple Primary Lung Adenocarcinomas

**DOI:** 10.32604/or.2026.078309

**Published:** 2026-05-21

**Authors:** Dat Quoc Tran, Mayu Takeda, Eiji Sugihara, Tetsuya Tsukamoto, Yasushi Hoshikawa, Yasuyoshi Mizutani, Kazuya Shiogama, Naoya Asai, Atsuko Niimi, Makoto Sumitomo, Hideyuki Saya, Motoshi Suzuki

**Affiliations:** 1 Department of Molecular Oncology, Fujita Health University, Toyoake, Japan; 2 Oncology Innovation Center, Fujita Health University, Toyoake, Japan; 3 Department of Diagnostic Pathology, Fujita Health University, Toyoake, Japan; 4 Department of Thoracic Surgery, Fujita Health University, Toyoake, Japan; 5 Department of Pathology and Cytopathology, Fujita Health University, Toyoake, Japan; 6 Department of Pathology, Fujita Health University, Toyoake, Japan

**Keywords:** Multiple primary lung cancer, intrapulmonary metastasis, *BRCA2 DNA repair associated*, germline pathogenic variants, adenocarcinoma

## Abstract

**Objectives**: Genetic risk models have substantially advanced our understanding of germline pathogenic variants (GPVs) in some malignancies, whereas their clinical significance in lung cancer remains unclear. The present study aimed to better understand potential contribution of GPVs to lung cancer etiology. **Methods**: A targeted sequencing panel of 143 cancer-related genes was applied to analyze 26 distinct lung adenocarcinoma (LUAD) tumors from 11 patients histopathologically diagnosed with multiple primary lung cancers (MPLC). Tumor classification was performed through integrated evaluation of mutation profiles, and variants shared among tumor lesions were further validated as likely germline or somatic mutations using Sanger sequencing. **Results**: Mutation profiles were compared to reveal clonal relationships among lesions in each patient. Nine of the 11 cases (81.8%) were classified as MPLC, 1/11 (9.1%) as intrapulmonary metastasis (IM), and 1/11 (9.1%) exhibited features of both MPLC and IM. Among the nine MPLC cases, eight (88.9%) harbored matching variants across independent tumor lesions that were also detected in tumor-adjacent regions, suggesting classification as likely germline variants. Importantly, among the eight cases with shared variants, one possessed a novel truncating *BRCA2 DNA repair associated* (*BRCA2*) variant (p.N900IfsTer4), while the others harbored variants of uncertain significance (VUS) in the *tumor protein p53* (*TP53*), *caspase recruitment domain family member 11* (*CARD11*), *platelet derived growth factor receptor beta* (*PDGFRB*), *lysine methyltransferase 2D* (*KMT2D*), *phosphoinositide-3-kinase regulatory subunit 1* (*PIK3R1*), *neuregulin 1* (*NRG1*), *androgen receptor* (*AR*), and *KIT proto-oncogene*, *receptor tyrosine kinase* (*KIT*) genes. To determine whether a similar *BRCA2* variant was present in other lung cancer patients, 123 LUAD cases were analyzed, and one (0.81%) possessing a truncating *BRCA2* variant (p.Q1429FfsTer20) without any typical driver mutations was identified. **Conclusions**: *BRCA2* GPVs may represent putative pathogenic mutations, and thus be potential molecular targets for future treatment of LUAD.

## 1 Introduction

According to the GLOBOCAN 2022 database, lung cancer remains the leading cancer worldwide in regard to both incidence and mortality [1]. Recent advances in imaging technology and histopathology have resulted in increasing numbers of patients diagnosed with multiple lung cancers (MLC), defined by the presence of two or more independent malignant lesions in the lungs, occurring either synchronously or metachronously [2,3,4]. Accurate distinction between multiple primary lung cancers (MPLC) and intrapulmonary metastasis (IM) is crucial, as it directly affects disease staging and treatment strategies [5,6,7,8]. For comparing genomic profiles across tumor loci, several methods based on Next-Generation Sequencing (NGS) technology have recently been developed [9,10,11]. Among those, whole-genome sequencing (WGS) and whole-exome sequencing (WES) provide comprehensive genomic information for better discrimination of MPLC from IM [12,13], however, their use for determining the status of every tumor examined in routine clinical settings is impractical. Integration of targeted sequencing panels with histopathological evaluation has therefore become a promising approach, and its clinical utility warrants further investigation.

In addition to tumor classification, elucidation of the contribution of germline pathogenic variants (GPVs) to lung adenocarcinoma carcinogenesis is an important issue. In a study of primary lung cancers in 7788 patients, germline genetic testing identified enrichment of GPVs in *BRCA2 DNA repair associated* (*BRCA2*), *ATM serine*/*threonine kinase* (*ATM*), *checkpoint kinase 2* (*CHEK2*), *BRCA1 DNA repair associated* (*BRCA1*), and mismatch repair genes as compared with an unaffected control group [14]. Among high- or moderate-penetrance DNA damage repair genes, *BRCA2* exhibited the highest detection prevalence in lung cancer cases, especially those with lung adenocarcinoma (LUAD) [15]. Additionally, a recent study found that GPVs in *BRCA2* and *TP53* were associated with early-onset LUAD in an Asian population [16].

A large population-based genetic association study found that rare pathogenic variants in cancer predisposition genes were more strongly associated with multiple primary cancer diagnoses (2.6-fold greater) as compared with single primary cancer diagnoses (1.8-fold greater) [17]. Furthermore, enriched GPVs in *BRCA2*, *TP53*, and *RAD51D* have consistently been found in Asian patients with dual primary breast and lung malignancies, thus highlighting the potential contribution of genetic predisposition in multiple cancer etiology [18]. Although genetic risk models have substantially advanced our understanding of GPVs in several malignancies, including breast, ovarian, prostate, and pancreatic cancers [19,20,21,22], their clinical significance in lung cancer remains unclear.

In this study, we aimed to analyze tumors from patients with multiple lung cancer adenocarcinoma by integrating histopathological and molecular classification, and to identify putative germline pathogenic variants in cancer susceptibility genes that may contribute to lung cancer etiology, with particular focus on *BRCA2*.

## 2 Materials and Methods

### 2.1 Patient Selection

From June 2016 to December 2020, 699 patients were diagnosed with lung cancer and subsequently underwent surgical treatment, either synchronously or metachronously, at Fujita Health University Hospital (Aichi, Japan). Among 53 patients with multiple lung adenocarcinomas, 11 were diagnosed as MPLC by histopathological evaluation and selected for this study, with a total of 26 distinct tumor samples being employed for NGS analysis (Table S1). Furthermore, 125 LUAD samples obtained from 123 individuals examined at Fujita Health University Hospital between July 2021 and March 2023 were also analyzed. The present study was conducted following approval from the Ethics Committee of Fujita Health University (No. HM22-264, HM22-506, and HM25-017), and written informed consent was obtained from each participant.

### 2.2 Sample Preparation and Sequencing Analysis

Genomic sequencing was performed using the PleSSision-Rapid Neo testing platform, as previously described [23]. The panel included 143 cancer-related genes, targeting the full coding sequences of each gene. Briefly, sections on hematoxylin- and eosin-stained slides were marked as tumor or normal by the examining pathologist. Genomic DNA was extracted from 10-mm-thick formalin-fixed paraffin-embedded (FFPE) tissue sections of tumor specimens using the QIAamp DNA FFPE Tissue Kit (cat# 56404, Qiagen, Germany), following the manufacturer’s instructions. The quality of sample, defined by a DNA integrity number (DIN) > 2.0, and DNA concentration were determined using a 4200 TapeStation System (Agilent Technologies, Germany). Samples with high-quality DNA were used for sequencing with a 143-gene targeted panel using the NextSeq 2000 system (Illumina, San Diego, California, USA) (Table S2). Variant annotation and classification were performed using the GenomeJack bioinformatics pipeline (Mitsubishi Electric Software Corporation, Japan), as previously described [24]. All variants were aligned to the human reference genome GRCh37 (hg19).

### 2.3 Determination of Somatic and Germline Status of Matching Variants

Following extraction of genomic DNA from cancer regions and corresponding normal tissues from each tumor lesion, target loci were amplified by PCR (primer sequences presented in Table S3), followed by Sanger sequencing. Data obtained were analyzed using the Sequencing Scanner software package, version 2 (Applied Biosystems, Foster City, California, USA). Variants were classified as somatic when present only in tumor tissues, whereas those present in both a tumor and corresponding normal tissue were classified as likely germline [25]. Variant annotations and classifications were performed with reference to the human genomic databases COSMIC (https://cancer.sanger.ac.uk/cosmic), ClinVar (https://www.ncbi.nlm.nih.gov/clinvar), CIViC (https://civicdb.org/home), Clinical Knowledgebase (CKB) (https://ckb.genomenon.com), Japanese SNP database, version 2.3 (https://www.hgvd.genome.med.kyoto-u.ac.jp), Japanese SNV frequency data (https://jmorp.megabank.tohoku.ac.jp), and The Exome Aggregation Database, version 4.1.0 (https://gnomad.broadinstitute.org). Variants were classified as pathogenic driver mutations if they were registered as pathogenic in at least one of the referenced databases. All database assessments were completed by 30 November 2025. Classification of the novel *BRCA2* likely germline variants was performed according to the American College of Medical Genetics and Genomics, and the Association for Molecular Pathology (ACMG/AMP) guidelines [26].

### 2.4 Hematoxylin-Eosin and TTF-1 Staining

All sections were stained with hematoxylin (Muto Pure Chemicals, cat# 32042, Tokyo, Japan)-eosin (Muto Pure Chemicals, cat# 30141, Tokyo, Japan) and processed for immunohistochemistry. For immunohistochemical analysis, the sections were deparaffinized with xylene and rehydrated through graded ethanol. Endogenous peroxidase was quenched with 0.03% hydrogen peroxide in methanol for 30 min at room temperature. Heat-induced epitope retrieval was applied using a pressure pan (Delicio 6L, T-FAL, Rumily, France) for 10 min. Preliminary experiments determined 1 mmol/L ethylenediamine tetraacetic acid solution, pH 8.0, for the optimal soaking solution for heating. After pressure cooking, the sections were left for cooling in the soaking solution for 30 min. Anti-Thyroid Transcription Factor-1 (TTF-1) mouse monoclonal antibody (Dako, cat# M3575, Glostrup, Denmark) was incubated overnight at room temperature. After rinsing in 10 mmol/L phosphate-buffered saline, pH 7.2, the sections were incubated with the universal immunoperoxidase polymer, anti-mouse and -rabbit (Histofine Simple Stain MAXPO; Nichirei, cat# 424151, Tokyo, Japan) at room temperature for 30 min. The reaction products were visualized in Liquid DAB+ substrate chromogen system (Dako, cat# K3468, Glostrup, Denmark). The nuclei were lightly counterstained with Mayer’s hematoxylin.

### 2.5 Statistical Analysis

No statistical analysis was included in this study.

## 3 Results

### 3.1 Mutation Profiling

**Figure 1:**
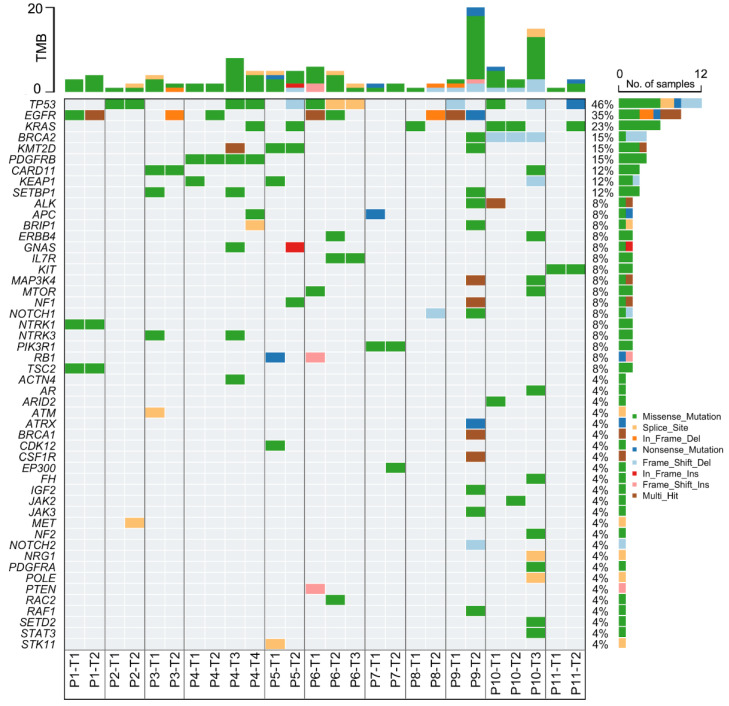
Oncoplot heatmap showing variants in 26 tumors from 11 patients with multiple lung cancers, indicating the top 50 genes ranked based on mutation frequency from high to low. Each cell in the plot is color-coded based on mutation type. Stacked bar charts in upper and right panels show tumor mutation burden (TMB) and mutation type frequency for each gene, respectively. Tumor sample IDs are presented at the bottom.

**Figure 2:**
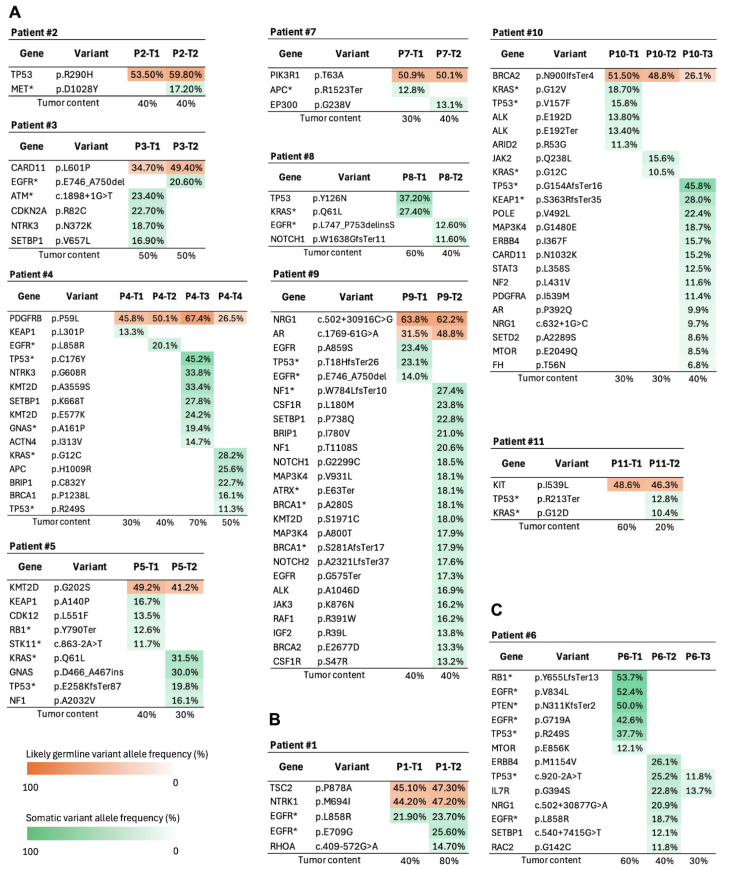
Gene variation profiles of 11 multiple lung cancer patients. (**A**) Nine patients were classified as MPLC, (**B**) one as IM, and (**C**) one as a combination of MPLC and IM. Each table lists variants detected for each tumor lesion, along with corresponding allele frequencies. Tumor content for each lesion is presented at the bottom. Likely germline and somatic variant allele frequencies are depicted in orange and green, respectively. *Gene with pathogenic driver mutations.

This study was conducted to identify variants associated with potential driver activities. Sequencing of adenocarcinoma tumors from 11 MLC patients was performed, including eight patients with two, two with three, and one with four independent lesions (Table S1, Fig. 1). The molecular profile of each tumor lesion is shown in Fig. 2. Among the 26 tumor samples, 124 variants were detected, with an average of 4.8 variants per tumor (range 2-25 per tumor). The most frequently mutated genes were *TP53* (46%, 12/26) and *EGFR* (35%, 9/26), followed by *KRAS* (23%). *TP53* showed a diverse spectrum of mutation and variant types, while missense and in-frame mutations were predominant in *EGFR*. Additional variants were identified in several oncogenes (*SETBP1*, *PDGFRB*, *CARD11*), tumor suppressor genes (*KMT2D*, *NRG1*, *KEAP1*), and homologous recombination deficiency-related genes (*BRCA1*, *BRCA2*), either within the same or across different tumor lesions from the same patients.

### 3.2 Identification of Matching Likely Germline Variants among Tumor Foci in MLC Cases by Targeted Sequencing

**Figure 3:**
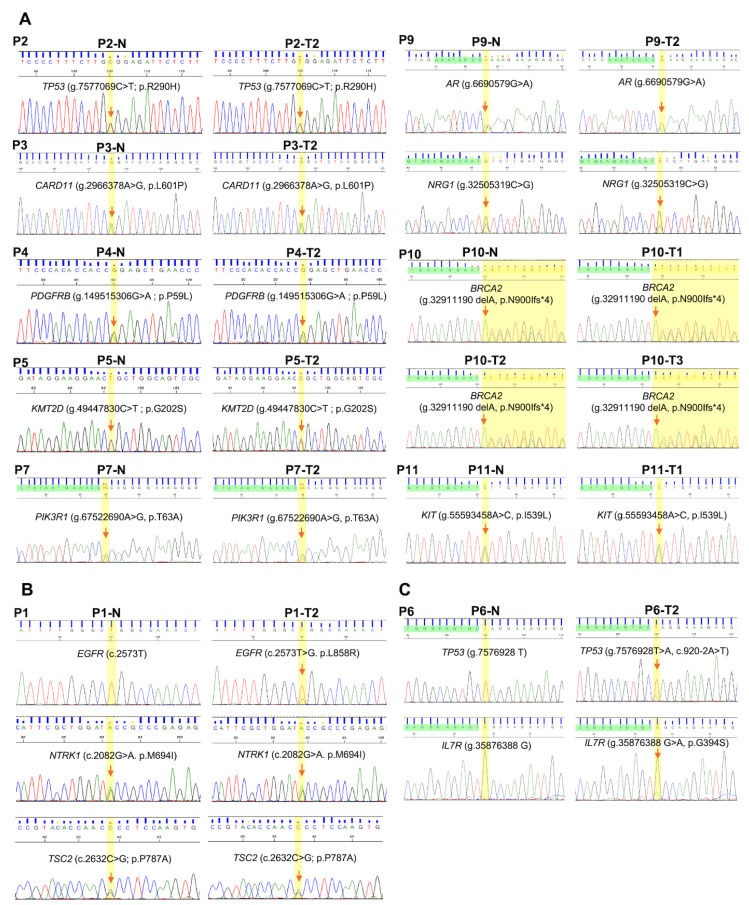
Verification of shared likely germline variants across tumor lesions by Sanger sequencing in (**A**) nine patients classified as MPLC, (**B**) one patient classified as IM, and (**C**) one patient classified as a combination of MPLC and IM. N: normal tissue, T: tumor tissue. Red arrows and yellow highlighted regions indicate variant positions in each gene.

Mutation profiling of all 11 MLC patients revealed that 10 harbored at least one shared variant between the tumor foci, with seven showing one, two showing two, and one showing three variants (Fig. 2). To determine whether these variants were somatic or germline, genomic DNA was extracted from corresponding cancerous and tumor-adjacent regions, and subjected to Sanger sequencing. Among the 14 shared variants, 11 were identified as likely germline (Fig. 2A,B and Fig. 3A,B). In contrast, the lung cancer driver mutation *EGFR* (p.L858R) in patient #1 (Fig. 2B and Fig. 3B), as well as a pathogenic *TP53* mutation (c.920-2A>T) and an *IL7R* variant of uncertain significance (VUS) (p.G394S) in patient #6 (tumors T2 and T3) were identified as somatic alterations (Fig. 2C and Fig. 3C).

### 3.3 Tumor Classification Based on Mutation Profile Evaluation

Based on the mutation profiles, nine cases were classified as MPLC, one as IM, and one as a combination of MPLC and IM (Fig. 2, Table 1).

**Table 1:** MLC patient outline.

Patient Group	No. of Cases	Subgroup (No. of Cases)
MPLC	9	-
with shared likely germline variants*	-	8
without shared likely germline variants	-	1
IM	1	-
Mix of MPLC and IM	1	-
**Total**	**11**	**9**

Note: *Number includes truncating BRCA2 DNA repair associated (BRCA2) variant. MLC: multiple lung cancer; MPLC: multiple primary lung cancer; IM: intrapulmonary metastasis.

**Figure 4:**
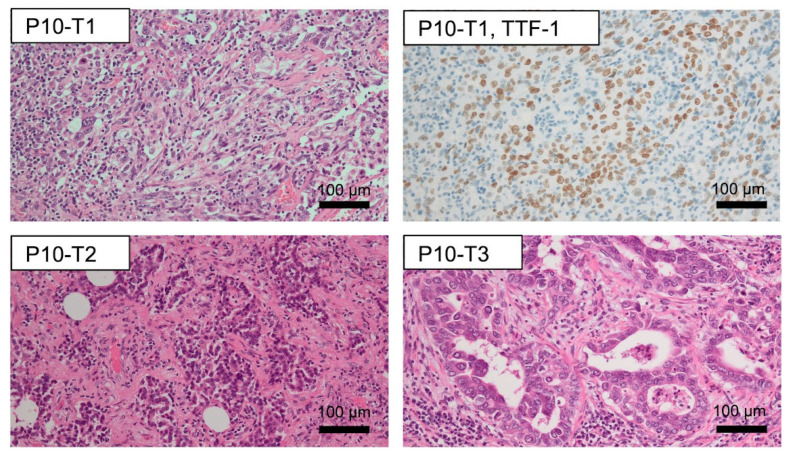
Histopathological features of tumor lesions from patient #10. Tumor lesion T1 was positive for TTF-1. Scale bar = 100 μm. P10-T1: Patient #10—Tumor lesion #1; P10-T2: Patient #10—Tumor lesion #2; P10-T3: Patient #10—Tumor lesion #3; TTF-1: Thyroid Transcription Factor-1.

Among the MPLC cases, patient #10 was found to have three tumors, with two foci located in the left upper and one in the right upper lobe (Table S1). The first tumor in patient #10 (P10-T1) was a TTF-1-positive solid adenocarcinoma with *KRAS* G12V and *TP53* V157F, while P10-T2 was 70% acinar and 30% papillary with *KRAS* G12C, and P10-T3 was 50% papillary, 30% acinar, and 20% solid with *TP53* and *KEAP1* truncations (Fig. 4, Table S1).

Notably, all tumors in this patient shared the *BRCA2* p.N900IfsTer4 likely GPV. Examinations of several databases for comparison indicated that this *BRCA2* alteration has not been previously reported (Table 2). In addition to *BRCA2*, several shared likely germline variants in *TP53*, *CARD11*, *PDGFRB*, *KMT2D*, *PIK3R1*, *NRG1*, *AR*, and *KIT* were detected in the MPLC cases.

**Table 2:** Variant annotations and multi-database classification of shared likely germline variants in multiple primary lung cancers (MPLC) cases.

Gene	Gene Function	Genomic Alteration	Transcription/cDNA Alteration	Amino Acid Change	ClinVar	CIViC	JAX CKB	HGVD	ToMMo	ExAC	dbSNP ID	COSMIC ID
TP53	TSG	g.7577069C>T	NM_000546.6: c.461delG	p.R290H	Benign	N/A	no effect	N/A	N/A	0.0%	rs55819519	COSM44017
CARD11	OG	g.2966378A>G	NM_032415.7: c.3096C>A	p.L601P	N/A	N/A	N/A	N/A	N/A	N/A	rs1303029402	N/A
PDGFRB	OG	g.149515306G>A	NM_002609.4: c.176C>T	p.P59L	VUS	N/A	N/A	N/A	N/A	0.0%	rs202213873	COSM5368833
KMT2D	TSG	g.49447830C>T	NM_003482.4: c.604G>A	p.G202S	Benign	N/A	N/A	N/A	N/A	N/A	rs1364190219	COSM124454
PIK3R1	TSG	g.67522690A>G	NM_181523.3: c.187A>G	p.T63A	N/A	N/A	N/A	N/A	N/A	N/A	N/A	N/A
NRG1	TSG	g.32505319C>G	NM_013956.5: c.502+30916C>G	-	N/A	N/A	N/A	N/A	N/A	N/A	rs1563846753	N/A
AR	OG	g.66905791G>A	NM_000044.6: c.1769-61G>A	-	Likely benign	N/A	N/A	N/A	N/A	N/A	rs200700978	N/A
BRCA2	HRD	g.32911190del	NM_000059.4: c.2698delA	p.N900IfsTer4	N/A	N/A	N/A	N/A	N/A	N/A	N/A	N/A
KIT	OG	g.55593458A>C	NM_000222.3: c.1615A>C	p.I539L	VUS	N/A	N/A	N/A	N/A	N/A	N/A	N/A

Note: HRD: homologous recombination deficiency; TSG: tumor suppressor gene; OG: oncogene. ClinVar: public database of relationships among human variations and phenotypes; CIViC: Clinical Interpretation of Variants in Cancer; JAXCKB: Jackson Laboratory Clinical Knowledgebase; HGVD: Japanese SNP database; ToMMo: Japanese SNV frequency data; ExAC: The Exome Aggregation Consortium; dbSNP: NCBI database of genetic variations; COSMIC: Catalog Of Somatic Mutations In Cancer. VUS: variant of uncertain significance. N/A: not available.

Based on the presence of shared driver somatic mutations and additional mutation patterns in patient #1, P1-T2 was classified as an IM lesion originating from P1-T1. Specifically, P1-T1 and P1-T2 shared a somatic *EGFR* driver mutation (p.L858R), as well as likely germline variants in *TSC2* (p.P878A) and *NTK1* (p.M694I) (Fig. 2B and Fig. 3B). Tumor P1-T2 showed more mutations than P1-T1, with an additional *EGFR* p.E709G driver mutation, suggesting that P1-T1 was the primary tumor.

Patient #6 had three tumors (Table S1). P6-T1 shared no somatic mutations with the other lesions, suggesting that it was independent. In contrast, P6-T2 and P6-T3 shared a pathogenic somatic *TP53* mutation (c.920-2A>T) along with a VUS in *IL7R* (p.G394S) (Fig. 2C and Fig. 3C). Since P6-T2 harbored more mutations than P6-T3, it was considered that P6-T2 and P6-T3 were IM and primary tumors, respectively.

### 3.4 Detection of BRCA2 Variants in the Second Lung Adenocarcinoma Patient Cohort

To evaluate whether any *BRCA2* variants were present in LUAD cases, 125 independent LUAD tumors obtained from 123 individuals treated at our hospital were sequenced using the same sequencing panel. Tumor mutation profiling identified that a patient possessing no apparent driver mutations harbored a truncating *BRCA2* variant (p.Q1429FfsTer20) with a tumor variant allele frequency of 50.4%, suggesting a germline alteration. Examinations of several databases indicated that this *BRCA2* alteration has not been previously reported (Table 3).

**Table 3:** Analysis of the 125 lung adenocarcinoma (LUAD) tumors identified one patient harboring an additional truncating *BRCA2 DNA repair associated* (*BRCA2*) variant.

Patient ID	Gene	Gene Function	cDNA Alteration	Amino Acid Change	Tumor VAF (%)	ClinVar	CIViC	JAX CKB	HGVD	ToMMo	ExAC	dbSNP ID	COSMIC ID
LUAD-1	BRCA2	TSG	c.4278_4279insTT	p.Q1429FfsTer20	50.4	N/A	N/A	N/A	N/A	N/A	N/A	N/A	N/A
	EPCAM	other	c.754G>C	p.V252L	49.3	VUS	N/A	N/A	N/A	N/A	0%	rs773185578	N/A
	MET	OG	c.2942-13_2960delTCTCTGTTTTAAGATCTGGGCAGTGAATTAGT	p.?	16.0	N/A	N/A	N/A	N/A	N/A	N/A	N/A	N/A
	ATM	TSG	c.2467-1G>T	p.?	5.6	N/A	N/A	N/A	N/A	N/A	N/A	N/A	N/A
	STAT3	OG	c.1481C>A	p.T494N	7.2	N/A	N/A	N/A	N/A	N/A	N/A	N/A	N/A

Note: TSG: tumor suppressor gene; OG: oncogene. ClinVar: public database of relationships among human variations and phenotypes; CIViC: Clinical Interpretation of Variants in Cancer; JAXCKB: Jackson Laboratory Clinical Knowledgebase; HGVD: Japanese SNP database; ToMMo: Japanese SNV frequency data; ExAC: The Exome Aggregation Consortium; dbSNP: NCBI database of genetic variations; COSMIC: Catalog of Somatic Mutations in Cancer. VUS: variant of uncertain significance. VAF: variant allele frequency. p.?: protein consequence undetermined. N/A: not available.

## 4 Discussion

Targeted panel sequencing for 26 tumors obtained from 11 patients diagnosed with multiple lung adenocarcinomas was performed for the present study. Tumors in nine of the patients exhibited distinct molecular profiles and were classified as MPLC, while those in one were classified as IM, and the final patient exhibited both IM and MPLC (Fig. 2). These results are in line with the International Association for the Study of Lung Cancer guidelines and other reports, which caution against classification of multiple lesions as IM or MPLC based solely on morphological analysis, and strongly recommend integrating molecular testing [27,28,29].

In addition to providing improved diagnostic precision, the present findings also highlight the potential contribution of shared predisposition VUS among patient lesions, which may underlie the development of multiple independent tumors. Several germline variants were identified across multiple tumor foci, including alterations in the homologous recombination gene *BRCA2*, tumor suppressor genes *TP53*, *KMT2D*, *PIK3R1*, and *NRG1*, and oncogenes *CARD11*, *PDGFRB*, *AR*, and *KIT* (Table 2). Currently, the contribution of VUS to multiple lung cancer development and their clinical relevance remain unclear due to insufficient data to assess pathogenicity. However, it is possible that these germline variants cumulatively contribute to tumor development. Additional functional studies and computational predictions are required to determine their clinical significance.

**Figure 5:**
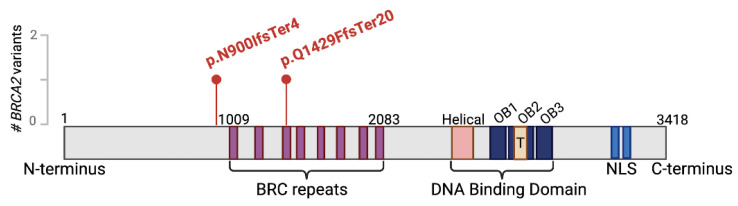
Lollipop plots illustrating predicted distribution of two novel likely germline pathogenic variants (GPVs) in *BRCA2 DNA repair associated* (*BRCA2*) gene. Amino acid changes are indicated for each variant. The plots were generated using BioRender (reference genome: GRCh37, GenBank reference BRCA2: NM_000059).

Two novel truncating *BRCA2* variants were detected in the present study. The first variant (p.N900IfsTer4) was found across three tumor lesions from an MPLC case. Screening of the second LUAD cohort identified one patient without any apparent driver mutations, though harboring a distinct truncating *BRCA2* variant (p.Q1429FfsTer20). Protein domain mapping revealed the location of p.N900IfsTer4 upstream of the BRC repeat region (amino acid 1009-2083), while p.Q1429FfsTer20 was found within BRC repeat 3, a critical domain essential for RAD51 binding (Fig. 5). The p.N900IfsTer4 variant truncates BRCA2 upstream of all eight BRC repeats and the C-terminal DNA-binding domain, indicating a complete loss-of-function effect. In contrast, the p.Q1429FfsTer20 variant, though retaining the BRC repeats 1 and 2, results in loss of BRC repeats 4–8 and downstream functional domains, potentially leading to homologous recombination deficiency. According to the ACMG/AMP guidelines, these frameshift variants fulfill the PVS1 criterion for a null variant in a well-established tumor suppressor gene. Additionally, both variants satisfy the PM2 criterion, as they are absent from control population databases, including the Exome Aggregation Consortium and gnomAD v4.1, thereby supporting their classification as likely pathogenic variants [26]. Such *BRCA2* germline alterations likely result in a haploinsufficient phenotype and may be associated with an increased risk of oncogenesis.

The effects of pathogenic *BRCA2* mutations, which impair the efficacy of DNA damage response (DDR), on lung cancer development remain controversial, though some studies have reported findings showing an association of *BRCA2* GPVs with a high risk of lung cancer [15,16,30]. A large case-control study that included 63,828 patients with 14 common cancer types and 37,086 controls found that the odds ratio for lung cancer was 1.7-fold greater, though it did not reach a level of statistical significance [31]. These findings highlight the need for further investigation of the contribution of *BRCA2*-mediated DDR deficiency, not only to the risk of MPLC development but also to the pathogenesis of primary LUAD.

In addition to the carcinogenic contribution of *BRCA2* GPVs to LUAD, an increasing clinical concern is whether these tumors exhibit functional homologous recombination deficiency (HRD), which may predict sensitivity to PARP inhibitors. While our approach identified likely germline *BRCA2*-mediated HRD, further investigation, such as analysis of mutational signatures or functional analyses, is essential to confirm HRD. Thus patients carrying these variants may have opportunities to benefit from PARP inhibitor therapy [32,33], platinum-based chemotherapy [34], nanomedicine-based delivery strategies [35,36,37], and other molecular reprogramming approaches [38].

Recently, precision oncology frameworks that integrate somatic and germline mutation data with genetic counselling have significantly improved diagnostic workflows and personalized care, particularly for LUAD patients with multiple primary tumors or lacking driver mutations [39]. In terms of diagnostic application, NGS analysis using both tissue and liquid biopsy samples may increase detection of somatic and germline variants, thereby enabling comprehensive genomic profiling, enhancing diagnostic precision, and providing additional information for clinical management of NSCLC patients [40]. From a therapeutic perspective, germline genomic profiling can inform treatment selection and classify patients for clinical trials aimed at developing new personalized therapeutic approaches [41].

Nevertheless, the present study has several limitations. First, use of tumor-adjacent regions as normal controls is generally not recommended for confirming germline variants because of various cancer-related phenomena, including possible field effects [42]. As in other retrospective analyses involving deceased patients, the lack of peripheral blood or other normal tissue samples limited our ability to definitively confirm germline status. Furthermore, the present cohorts were relatively small, that have only 11 MPLC and 123 LUAD patients. This may explain why the observed *BRCA2* variant frequency in the LUAD cohort (0.81%) was slightly higher than that reported for the general population (0.1-0.4%) [43]. The present study also lacked functional assays to demonstrate HRD in patients with BRCA2 GPVs.

## 5 Conclusion

This study identified putative PGVs, including two novel truncating *BRCA2* variants, which provide insight into molecular mechanisms involved in lung adenocarcinoma development. Moreover, it is considered that these likely germline variants could serve as novel targets for therapeutic strategies in primary and multiple primary LUAD cases, as well as for early detection in high-risk healthy individuals.

## Data Availability

Data for the MPLC cohort in this study are included within the article and Supplementary Materials. Data for the additional primary lung adenocarcinoma cohort can be obtained upon reasonable request by contacting the corresponding author via email.

## Supplementary Materials

The supplementary material is available online at https://www.techscience.com/doi/10.32604/or.2026.078309/s1.

## Abbreviations

DDR	DNA damage response
GPV	Germline pathogenic variant
HRD	Homologous recombination deficiency
IM	Intrapulmonary metastasis
LUAD	Lung adenocarcinoma
MLC	Multiple lung cancers
MPLC	Multiple primary lung cancers
OG	Oncogene
TSG	Tumor suppressor gene
VUS	Variant of uncertain significance
