# Supplementary material for: Identification of BRCA2 Likely Germline Pathogenic Variants in Patients with Multiple Primary Lung Adenocarcinomas
Source: Oncol Res. 2026 May 21;34(6):15. doi: 10.32604/or.2026.078309 (PMC13223183; doi:10.32604/or.2026.078309)
Supplement: Supplementary file 1 [file OncolRes-34-78309-s001.zip › TSP_OR_78309-s001.docx]

**Supplementary Materials**

**Table S1:** Clinical characteristics and tumor histopathological subtypes

|  | **Tumor ID** | **Age at diagnosis** | **Sex** | **Tumor location** | **Histology** | **Pathological subtype** | | | | |
| --- | --- | --- | --- | --- | --- | --- | --- | --- | --- | --- |
|  |  |  |  |  |  | **Solid (%)** | **Acinar (%)** | **Lepidic (%)** | **Papillary (%)** | **Micropapillary (%)** |
| Patient #1 | P1-T1 | 73 | F | RML | Pure GGN | 0 | 0 | 0 | 0 | 0 |
|  | P1-T2 | 71 | F | LUL | BAC | 0 | 0 | 0 | 0 | 0 |
| Patient #2 | P2-T2 | 74 | F | RUL | N/A | 0 | 10% | 30% | 60% | 0 |
|  | P2-T2 | 74 | F | RLL | N/A | 0 | 20% | 30% | 50% | 0 |
| Patient #3 | P3-T1 | 73 | F | RUL | N/A | 0 | 0 | 10% | 90% | 0 |
|  | P3-T2 | 73 | F | RML | AIS | 0 | 0 | 0 | 0 | 0 |
| Patient #4 | P4-T1 | 70 | M | LUL | N/A | 0 | 70 | 30 | 0 | 0 |
|  | P4-T2 | 70 | M | LUL | N/A | 0 | 60 | 40 | 0 | 0 |
|  | P4-T3 | 70 | M | RUL | N/A | 0 | 20 | 0 | 80 | 0 |
|  | P4-T4 | 70 | M | RUL | N/A | 0 | 0 | 20 | 80 | 0 |
| Patient #5 | P5-T1 | 82 | M | LUL | N/A | 0 | 85 | 0 | 10 | 5 |
|  | P5-T2 | 82 | M | RLL | N/A | 10 | 0 | 0 | 90 | 0 |
| Patient #6 | P6-T1 | 66 | F | LUL | N/A | 50 | 15 | 15 | 20 | 0 |
|  | P6-T2 | 66 | F | LUL | N/A | 0 | 60 | 10 | 30 | 0 |
|  | P6-T3 | 66 | F | LUL | AIS | 0 | 0 | 0 | 0 | 0 |
| Patient #7 | P7-T1 | 76 | M | LUL | N/A | 0 | 20 | 0 | 80 | 0 |
|  | P7-T2 | 75 | M | RUL | N/A | 0 | 95 | 5 | 0 | 0 |
| Patient #8 | P8-T1 | 75 | M | RUL | N/A | 0 | 10 | 30 | 60 | 0 |
|  | P8-T2 | 72 | M | LUL | N/A | 0 | 0 | 0 | 100 | 0 |
| Patient #9 | P9-T1 | 72 | F | RUL | N/A | 0 | 90 | 10 | 0 | 0 |
|  | P9-T2 | 72 | F | RUL | N/A | 0 | 0 | 10 | 90 | 0 |
| Patient #10 | P10-T1 | 56 | F | LUL | N/A | 100 | 0 | 0 | 0 | 0 |
|  | P10-T2 | 56 | F | LUL | N/A | 0 | 70 | 0 | 30 | 0 |
|  | P10-T3 | 55 | F | RUL | N/A | 20 | 30 | 0 | 50 | 0 |
| Patient #11 | P11-T1 | 56 | F | LLL | N/A | 0 | 20 | 70 | 10 | 0 |
|  | P11-T2 | 52 | F | LLL | mucinous | 0 | 0 | 0 | 0 | 0 |

F: female; M: male; RUL: right upper lobe; RML: right middle lobe; RLL: right lower lobe; LUL: left upper lobe; LLL: left lower lobe. N/A: not available

**Table S2:** PleSSision rapid panel showing 164 cancer-related genes for SNV detection

| *ABL1* | *ACTN4* | *AKT1* | *AKT2* | *AKT3* | *ALK* | *APC* | *AR* | *ARAF* | *ARID1A* |
| --- | --- | --- | --- | --- | --- | --- | --- | --- | --- |
| *ARID2* | *ASXL1* | *ATM* | *ATRX* | *AXIN1* | *AXL* | *BAP1* | *BARD1* | *BCL2L11* | *BIRC3* |
| *BRAF* | *BRCA1* | *BRCA2* | *BRIP1* | *CARD11* | *CASP2* | *CCND1* | *CD274* | *CD79B* | *CDH1* |
| *CDK12* | *CDK4* | *CDKN2A* | *CHEK2* | *CNNM2* | *CREBBP* | *CRKL* | *CSF1R* | *CTNNB1* | *CUL3* |
| *DDR2* | *DNMT3A* | *ECT2L* | *EGFR* | *ENO1* | *EP300* | *EPCAM* | *ERBB2* | *ERBB3* | *ERBB4* |
| *ESR1* | *EZH2* | *FANCA* | *FANCD2* | *FANCE* | *FBXW7* | *FGFR1* | *FGFR2* | *FGFR3* | *FGFR4* |
| *FH* | *FLT3* | *GNA11* | *GNAQ* | *GNAS* | *GRIN2A* | *HRAS* | *HSD3B1* | *IDH1* | *IDH2* |
| *IGF1R* | *IGF2* | *IL7R* | *INSRR* | *JAK1* | *JAK2* | *JAK3* | *KDM6A* | *KDR* | *KEAP1* |
| *KIT* | *KMT2D* | *KRAS* | *LRRFIP2* | *MAP2K1* | *MAP2K2* | *MAP2K4* | *MAP3K1* | *MAP3K4* | *MDM2* |
| *MDM4* | *MET* | *MIR4673* | *MIR4728* | *MLH1* | *MSH2* | *MSH6* | *MTOR* | *MYC* | *MYCN* |
| *MYCNOS* | *MYD88* | *NF1* | *NF2* | *NFE2L2* | *NOTCH1* | *NOTCH2* | *NOTCH3* | *NPM1* | *NRAS* |
| *NRG1* | *NT5C2* | *NTRK1* | *NTRK2* | *NTRK3* | *PALB2* | *PBRM1* | *PDGFRA* | *PDGFRB* | *PIK3CA* |
| *PIK3R1* | *PIK3R2* | *PMS2* | *POLD1* | *POLE* | *PRKCI* | *PTCH1* | *PTEN* | *RAC1* | *RAC2* |
| *RAD51C* | *RAF1* | *RB1* | *REEP5* | *RET* | *RHOA* | *ROS1* | *RPL21P4* | *SDCCAG8* | *SETBP1* |
| *SETD2* | *SH2D2A* | *SLC7A8* | *SMAD4* | *SMARCA4* | *SMARCB1* | *SMO* | *SPOP* | *SRC* | *SRP19* |
| *STAT3* | *STK11* | *STT3A* | *SYNE1* | *TERT* | *TP53* | *TSC1* | *TSC2* | *TSPAN31* | *VHL* |
| *WT1* | *XPC* | *XYLB* | *ZNF276* |  |  |  |  |  |  |

**Table S3**: Primers used in this study

| **No** | **Target gene** | **Primer name** | **Supplier** | **Sequence (sense 5’-3’)** | **Product size (bp)** |
| --- | --- | --- | --- | --- | --- |
| 1 | *NTRK1* | NTRK1_M694I_F | SIGMA (custom) | TTTTTAATGATGGGGCTGGG | 157 |
|  |  | NTRK1_M694I_R |  | GTGGTGAACTTACGGTACAG |  |
| 2 | *TSC2* | TSC2_P878A_F | SIGMA (custom) | CACCTCTACAGGAACTTTGC | 185 |
|  |  | TSC2_P878A_R |  | AAAAACCGAGGCGCTCAG |  |
| 3 | *TP53* | TP53_R290H_F | SIGMA (custom) | GAGGCAAGGAAAGGTGATAA | 197 |
|  |  | TP53_R290H_R |  | CTTTGAGGTGCGTGTTTG |  |
| 4 | *CARD11* | CARD11_L601P_F | SIGMA (custom) | CAGACAGATGTGACCTCG | 185 |
|  |  | CARD11_L601P_R |  | GAGGCTAGCTACACTCTCC |  |
| 5 | *PDGFRB* | PDGFRB_P59L_F | SIGMA (custom) | AGTATTCTCCCGTGTCTAGC | 166 |
|  |  | PDGFRB_P59L_R |  | CTCAATGTCTCCAGCACC |  |
| 6 | *KMT2D* | KMT2D_G202S_F | SIGMA (custom) | AAAATCCAAGGCACATTTGG | 171 |
|  |  | KMT2D_G202S_R |  | GATGTCCACGGCTTTACC |  |
| 7 | *TP53* | TP53_920-2A-T_F | SIGMA (custom) | CGGCATTTTGAGTGTTAGAC | 185 |
|  |  | TP53_920-2A-T_R |  | CAGTTATGCCTCAGATTCAC |  |
| 8 | *IL7R* | IL7R_G394S_F | SIGMA (custom) | GGAAGAGATTCATCCCTCAC | 153 |
|  |  | IL7R_G394S_R |  | GTTTGTAGTCCCAAGGCTAA |  |
| 9 | *PIK3R1* | PIK3R1_T63A_F | SIGMA (custom) | AGCTCTTGGATTCAGTGATG | 199 |
|  |  | PIK3R1_T63A_R |  | AGTTTTCGAAGAACCTGGTG |  |
| 10 | *AR* | AR_1769-61G-A_F | SIGMA (custom) | TTTGGTGCCATACTCTGTC | 178 |
|  |  | AR_1769-61G-A_R |  | TAGTGCAATCATTTCTGCTG |  |
| 11 | *NRG1* | NRG1_502+30916C_F | SIGMA (custom) | CGATGGAGATTTATTCCCCA | 137 |
|  |  | NRG1_502+30916C_R |  | ATCTTCAGTCTGGGGCTC |  |
| 12 | *BRCA2* | BRCA2_N900Ifs_F | SIGMA (custom) | ACACAAATCTAAGAGTAATCCA | 198 |
|  |  | BRCA2_N900Ifs_R |  | GGTTCGTTTACACAAGTCAA |  |
| 13 | *KIT* | KIT_I539L_F | SIGMA (custom) | ATCCTGCCAAAGTTTGTGAT | 198 |
|  |  | KIT_I539L_R |  | TGTCTCAGTCATTAGAGCAC |  |
